# Clinical, Genetic, EEG, Neuroimaging Insights and Conservative Treatment in Pediatric Focal Epilepsy: A Retrospective Observational Study

**DOI:** 10.3390/jcm14072234

**Published:** 2025-03-25

**Authors:** Maria Cristina Gauci, Rosaria Gauci, Martino Ruggieri, Agata Polizzi, Andrea D. Praticò

**Affiliations:** 1 Postgraduate Training Program in Pediatrics, University of Catania, 95123 Catania, Italy; mariacristinagauci@gmail.com; 2 Unit of Pediatrics, Hospital Umberto I, 94100 Enna, Italy; 3 Unit of Pediatric Clinic, Department of Clinical and Experimental Medicine, University of Catania, 95123 Catania, Italy; a.polizzi1@unict.it; 4 Unit of Pediatrics, Department of Medicine and Surgery, University Kore of Enna, 94100 Enna, Italy

**Keywords:** focal epilepsy, EEG, MRI, pediatric epilepsy, focal cortical dysplasia, valproic acid, diagnostic imaging, seizure localization, epilepsy treatment, neurosurgical intervention

## Abstract

**Objective**: Focal epilepsy is the most frequent type of epilepsy in childhood, particularly after the first year of life. This study aims to analyze the clinical aspects, electrophysiological and neuroimaging findings, and genetic predispositions in pediatric focal epilepsy. Specifically, we investigate the association between these parameters and evaluate their impact on therapeutic decisions. **Methods**: This is a retrospective study, in which we enrolled 39 patients currently receiving follow-up in our unit, 20 male and 19 female. Using the Chi-squared test, we compared them considering several genetic traits, pre/peri/postnatal risk factors, family history, clinical and instrumental features, and treatments. Differences are considered significant with a *p* value < 0.005. **Results**: Our findings highlight the multifactorial nature of focal epilepsy, with a combination of genetic and environmental contributions. EEG demonstrated the highest sensitivity among diagnostic tools, being non-significant in only 12.8% of cases, while MRI (*p* < 0.001), CT (*p* < 0.04), and brain ultrasound had lower detection rates. MRI findings were significant in 43.6% of patients, predominantly showing vascular malformations (35.8%). MRI-negative findings were more common in temporal and occipital epilepsy, whereas MRI-positive results were observed in 100% of frontal seizures. Importantly, some MRI-negative cases may still be lesional, particularly in temporal lobe epilepsy, where focal cortical dysplasia could be present but undetected with standard imaging. Valproic acid remains the most commonly used anti-seizure medication, and, despite guideline recommendations, it was still prescribed as a first-line treatment in 34.3% of cases and is being used in 23.5% of female patients, raising concerns about its appropriateness. **Conclusions**: This study highlights the role of genetic and environmental risk factors in pediatric focal epilepsy. EEG showed superior diagnostic sensitivity over MRI, particularly in MRI-negative cases. While high-resolution MRI (3T or 7T) could improve lesion detection, its cost limits accessibility. Valproate was the most prescribed drug, despite its recommended use in generalized epilepsy, emphasizing the need for improved adherence to treatment guidelines. Together with other studies, these findings can contribute to optimizing diagnostic and therapeutic strategies for pediatric focal epilepsy.

## 1. Introduction

Epilepsy is a neurological disorder characterized by recurrent (≥2) unprovoked seizures occurring more than 24 h apart, as defined by the ILAE. Its causes include genetic, infectious, metabolic, immune, and structural factors, with some cases of unknown origin. The prevalence is 0.5–1%, with an incidence of 40–50 per 100,000 per year, higher in children and the elderly [1,2]. A focal seizure originates from a specific epileptic focus within one hemisphere, which may be highly localized or more widely distributed but remains regionally confined. The initial semiology of the seizure reflects or is consistent with the activation of a specific part of a cerebral hemisphere and is determined by the anatomo-functional properties of the involved structures. The progression of the seizure is characterized by the sequential and spatiotemporal integration of various elementary clinical manifestations, which may be positive or negative in nature, including motor, cognitive, sensory, autonomic, and other neurological symptoms. The discharge may secondarily generalize, affecting the contralateral hemisphere. Impairment of consciousness has no localizing value, as it depends on the extent of the ictal discharge and may be absent or variable. The interictal EEG typically reveals focal epileptiform discharges; however, diagnosis is primarily clinical, supported by EEG findings, neuroimaging, and genetic studies. In generalized epilepsy (the most frequent), the initial semiology of the discharge indicates or is consistent with an involvement of both cerebral hemispheres [2,3,4]. We herein present a retrospective longitudinal cohort study, recruiting children suffering from epilepsy of focal origin, diagnosed according to the new definition of the 2017 ILAE. Patients have been followed up in the pediatric wards of the University Hospital “Policlinico” of Catania and in the University Hospital “Umberto I” of Enna, Italy. This study aims to analyze seizure semiology, EEG patterns, and neuroimaging (MRI, CT, brain ultrasound) findings in pediatric focal epilepsy. We seek to determine associations between these parameters, evaluate the effectiveness of diagnostic tools in lesion detection, and compare our findings with existing literature to assess the impact of these investigations on therapeutic decisions.

## 2. Materials and Methods

This study was conducted following the STROBE (Strengthening the Reporting of Observational Studies in Epidemiology) guidelines to ensure transparency and reproducibility (see Supplementary Table S1). We initially selected 45 patients with a confirmed diagnosis of focal epilepsy. However, 6 patients were excluded due to inconsistencies between clinical diagnoses and EEG/MRI findings, or incomplete medical records. Our final study cohort consisted of 39 children, for whom we analyzed familial, genetic, clinical, diagnostic, and therapeutic aspects, including gender, age, specific genetic mutations, type of delivery, prenatal/perinatal/postnatal risk factors, seizure type, EEG abnormalities, presence of structural lesions on MRI, CT, or brain ultrasound (BU), and treatment type. The study protocol was chosen based on the need for a comprehensive, multidisciplinary assessment of pediatric focal epilepsy, integrating clinical, electrophysiological, neuroimaging, and genetic data. The retrospective observational design allowed for the analysis of real-world clinical practice.

The study was conducted in accordance with ethical guidelines, with approval from the Ethics Committee of the University Kore of Enna (n. 1983/2025). Informed consent was obtained from the parents or legal guardians of all participating children.

### 2.1. Inclusion and Exclusion Criteria

Patients were included if they met the following criteria: (1) a confirmed diagnosis of focal epilepsy based on clinical presentation and EEG findings, (2) availability of neuroimaging data (MRI, CT, or BU), (3) a minimum follow-up period of 12 months, and (4) completion of genetic testing.

Patients were excluded if they had (1) inconsistent clinical and diagnostic findings (e.g., discordance between EEG and MRI suggestive of alternative diagnoses), (2) incomplete or missing medical records that precluded accurate data analysis, (3) evidence of generalized epilepsy syndromes rather than focal epilepsy, or (4) prior epilepsy surgery. The exclusion of such cases ensured the reliability of the dataset and the validity of our findings.

### 2.2. Follow-Up and Diagnostic Approach

Patients were followed up for a minimum period of 12 months, with evaluations conducted every 3 to 6 months in specialized epilepsy clinics. The diagnosis of focal epilepsy was confirmed based on clinical history, seizure semiology, EEG findings, and neuroimaging assessments.

### 2.3. Seizure Etiology, Classification, and Characteristics

Seizures were classified according to the latest ILAE 2017 guidelines, distinguishing between focal awareness, focal impaired awareness, and focal to bilateral tonic–clonic seizures. Seizures were further categorized based on semiology into motor (e.g., tonic, clonic, myoclonic, automatisms, hyperkinetic) and non-motor (e.g., sensory, autonomic, behavioral arrest) manifestations. The etiology was classified as structural, genetic, metabolic, immune, infectious, or unknown, per ILAE criteria. Seizures were localized to specific lobes based on EEG and neuroimaging findings, distinguishing between temporal, frontal, parietal, and occipital lobe involvement. The classification and characterization of seizures followed standardized criteria to ensure diagnostic consistency across all patients. Drug-resistant epilepsy (DRE) was defined according to the latest ILAE criteria (2010 updated guidelines) as failure to achieve sustained seizure freedom after adequate trials of two appropriately chosen and tolerated anti-seizure medications (ASMs), used either as monotherapy or in combination. The classification of DRE included drug-resistant focal epilepsy with or without structural abnormalities, and cases were evaluated for potential surgical candidacy if seizures persisted despite medical management.

### 2.4. Clinical and Developmental Data Collection

A structured clinical interview was conducted with caregivers to obtain detailed prenatal, perinatal, and postnatal history, including exposure to risk factors such as maternal infections, fetal distress, and neonatal complications. Family history of epilepsy and neurological disorders was documented. Neurodevelopmental assessments were performed using standardized cognitive and behavioral screening tools, evaluating intellectual functioning, language development, and attention regulation. Behavioral and psychiatric comorbidities, including autism spectrum disorder (ASD) traits and ADHD, were assessed and recorded.

### 2.5. Neurophysiology Study

Electroencephalograms (EEGs) were performed in 39 patients diagnosed with focal epilepsy to assess for epileptiform abnormalities, disturbances in background activity, and other potential markers of cortical excitability. Standard 10–20-electrode placement was used, with recordings lasting at least 20 min, including wakefulness and, when possible, sleep stages. Video-EEG monitoring was conducted in selected cases presenting with clinically ambiguous events suggestive of seizures.

### 2.6. Neuroimaging

Magnetic resonance imaging (MRI) of the brain was performed in 39 patients using 1.5 Tesla scanners (GE Healthcare, Chicago, IL, USA) at the University Hospital “Policlinico” of Catania, Italy, following a standardized protocol that included T1-weighted, T2-weighted, FLAIR, DWI, and susceptibility-weighted imaging (SWI) sequences.

MRI assessments focused on identifying structural abnormalities associated with focal epilepsy, such as cortical malformations, hippocampal sclerosis, focal gliosis, and periventricular nodular heterotopia. Additionally, computed tomography (CT) scans and brain ultrasound (BU) were performed in 6 children, primarily in cases where MRI was contraindicated or in neonatal patients requiring urgent neuroimaging assessment. CT scans were evaluated for calcifications, hydrocephalus, or intracranial masses, while BU, performed through the anterior fontanelle in infants, assessed for ventricular dilation, hemorrhages, and midline structural abnormalities.

### 2.7. Genetics

Genetic testing was conducted for all patients, with next-generation sequencing (NGS) panels targeting epilepsy-related genes, as well as chromosomal microarray analysis (CMA) in selected cases. Whole-exome sequencing (WES) was performed in patients with atypical clinical presentations or additional neurologic features (e.g., chronic headache, movement disorders, intellectual disability).

### 2.8. Statistical Analysis

We used the STATA software, version 17 (developed by StataCorp LLC, College Station, TX, USA), which applies the Chi-squared test to compare variables. The *p*-value is considered not statistically significant if >0.05, significant if <0.05, and highly significant if <0.01.

## 3. Results

The study included 39 patients, consisting of 20 males (51.3%) and 19 females (48.7%). The mean age at seizure onset was 4.8 years (σ = 4.1 months). Seizures occurred before the age of one year in 28.2% of children, while 71.8% experienced their first seizure after the first year of life. Among these, 53.6% had seizure onset between one and five years of age, whereas 46.4% developed epilepsy at school age (≥6 years). A positive family history of seizures was noted in 38.5% of patients, primarily involving first- and second-degree relatives. When considering only direct family members, including parents, offspring, and siblings, the prevalence was 23.3%.

### 3.1. Prenatal, Perinatal, and Postnatal Risk Factors

**Figure 1:**
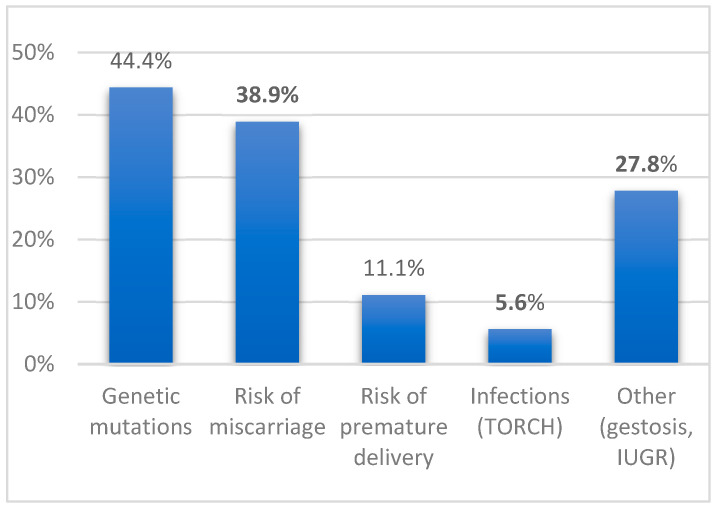
Prenatal risk factors. Legend: TORCH: Toxoplasma, Other Infections, Rubella, Cytomegalovirus, Herpes Virus. IUGR: Intrauterine Growth Restriction.

A history of dystocic delivery was observed in 56.4% of cases, while 41% were born through eutocic delivery, and 2.6% were born preterm. Prenatal risk factors were identified in 46.2% of patients (Figure 1). Genetic mutations were detected in a subset of these cases, with 50% involving mutations in the GNAQ gene (9q21), encoding the Gαq protein, which is associated with Sturge–Weber syndrome. Other mutations included those affecting genes associated with neurofibromatosis type 1 (NF1, 17q11.2), tuberous sclerosis (TSC1, 9q34), Aicardi syndrome (unspecified), and chromosomal abnormalities such as deletions involving TBL1XR1 (chromosome 3) and JARID2 (chromosome 6), a duplication on chromosome 16, and recurrent microduplications affecting 18 genes on chromosome 19.

**Figure 2:**
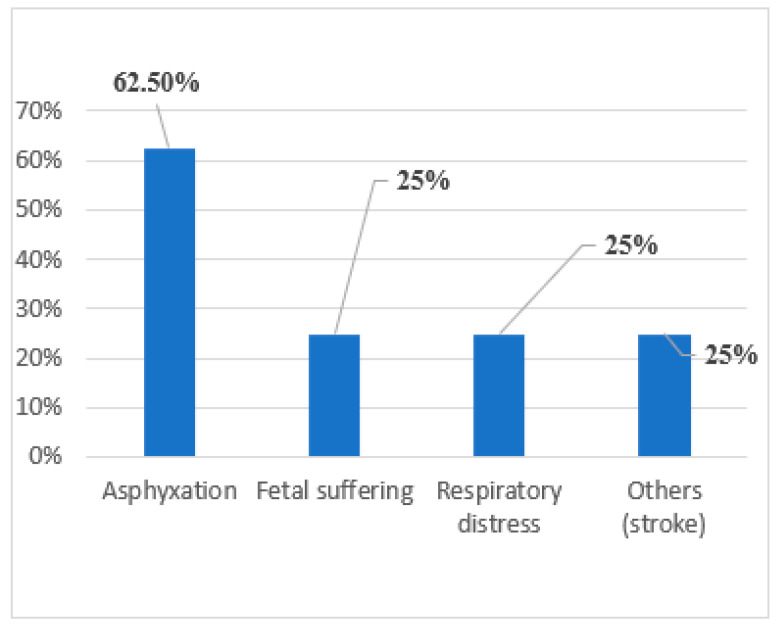
Perinatal risk factors.

Additional prenatal risk factors were documented in 27.8% of cases, including parental consanguinity, gestational hypertension (gestosis), intrauterine growth restriction (IUGR), congenital hypothyroidism, and fetal distress due to preterm labor. Perinatal complications that may have contributed to neurological damage were reported in 20.5% of children (Figure 2).

**Figure 3:**
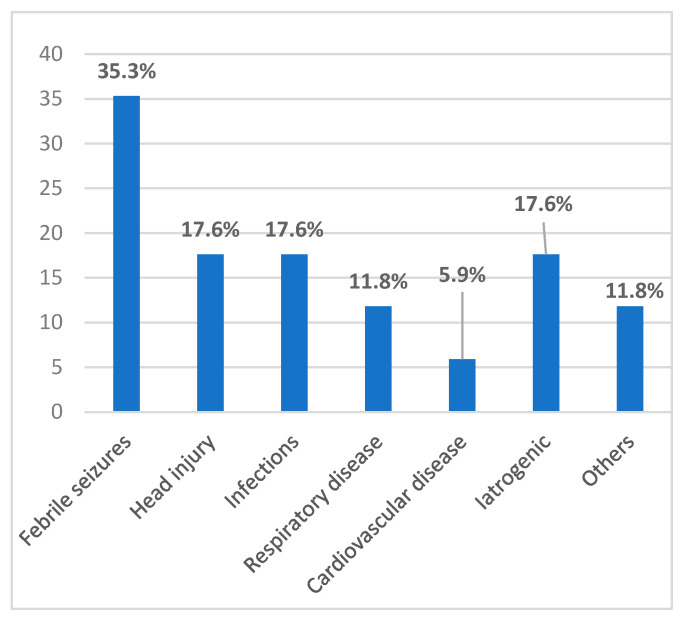
Postnatal risk factors.

A significant proportion of patients (43.6%) presented postnatal conditions favoring seizures (Figure 3), such as febrile seizures, head trauma, respiratory diseases including bronchiolitis and asthma, cardiac malformations associated with pulmonary hypertension, central nervous system infections, and specific therapeutic exposures such as chemotherapy, brain surgery, or long-term pharmacological treatments. Other contributing factors included acute disseminated encephalomyelitis (ADEM) and lack of vaccinations leading to central nervous system infections.

### 3.2. Clinical Aspects

**Figure 4:**
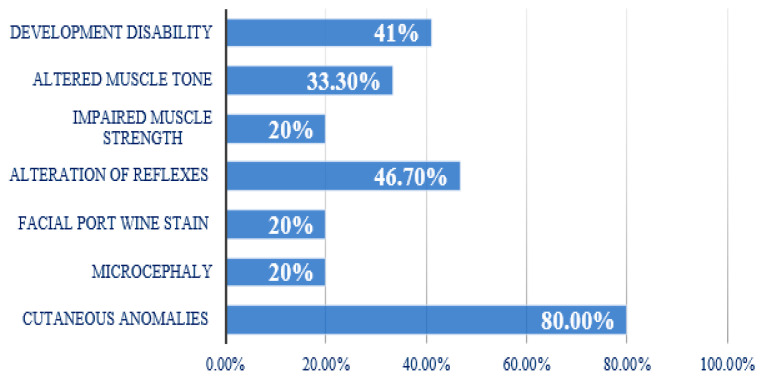
Clinical features other than epilepsy presented by the patients.

A thorough physical examination was conducted in all recruited children, with clinical abnormalities identified in 38.5% of cases. Among these, 80% exhibited cutaneous anomalies, predominantly café-au-lait spots, which were present in 91.7% of affected individuals, while 25% also had hypochromic macules. Microcephaly was observed in 20% of patients, whereas another 20% exhibited a facial port-wine stain, characteristic of Sturge–Weber syndrome. Reflex abnormalities were detected in 46.7% of cases, while 20% of patients had impaired muscle strength, and 33.3% showed altered muscle tone. Developmental disabilities were noted in 41% of children (Figure 4). A comprehensive summary of the clinical presentations of epileptic seizures, classified according to the focus involved, is provided in Table 1.

### 3.3. Electroencephalography

**Figure 5:**
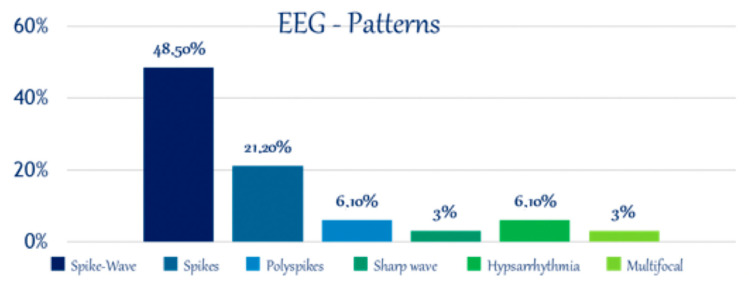
EEG patterns.

Electroencephalography (EEG) demonstrated abnormalities in 86.8% of cases, highlighting its effectiveness in detecting focal epileptic activity (Table 2). The temporal lobe was the most frequently affected region, observed in 42.1% of patients (16/38), followed by the central region in 28.9% (11/38), with 81.8% (9/11) of these cases also showing involvement of the temporal lobe. Occipital lobe activity was noted in 20.5% (8/38), while frontal lobe abnormalities were recorded in 7.7% (2/38). Distinct EEG discharge patterns included spike–wave complexes (48.5%), isolated spikes (21.2%), sharp waves (3%), polyspikes (3%), hypsarrhythmia (6.1%), and multifocal discharges (6.1%) (Figure 5).

**Figure 6:**
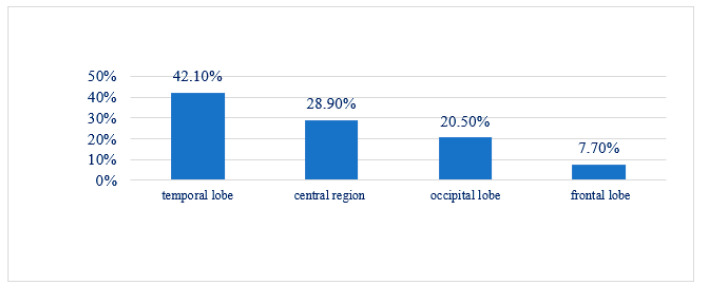
Epileptogenic focus.

Spike–wave complexes were predominantly linked to temporal lobe epilepsy (75%), followed by occipital epilepsy (62.5%) and frontal lobe epilepsy (100%). Isolated spikes were most frequently observed in the central region (71.4%) of cases (Figure 6).

**Table 2:** EEG and brain imaging of the patients.

Patient	EEG Findings	MRI Findings	CT Findings
1	Focal spikes (R, central)	Non-significant findings	Not performed
2	Normal	Normal	Not performed
3	Focal spike–wave discharges (R, temporal)	Subcortical tubers (R, temporal and frontal)	Not performed
4	Focal spikes (R, central)	Enlarged subarachnoid spaces	Not performed
5	Focal spike–wave (R, temporal)	Normal	Not performed
6	Normal	Normal	Not performed
7	Multifocal paroxysmal anomalies	Normal	Not performed
8	Focal spikes with secondary generalization (central and temporal)	Normal	Not performed
9	Sharp waves (L, central and temporal)	Normal	Not performed
10	Spike–wave (L, central and temporal)	Normal	Not performed
11	Generalized spike–wave (L, temporal and occipital; bilateral frontal)	Right mesial temporal sclerosis	Not performed
12	Generalized spike–wave (R, central and temporal)	Normal	Not performed
13	Spike–wave and polyspike–wave (R); hypsarrhythmia	Diffuse polymicrogyria, thickened and dysplastic right fronto-parietal cortex; hypoplasia of the corpus callosum (Figure 7)	Not performed
14	Normal	Enlarged subarachnoid space, cortical atrophy, microcalcifications, reduced vascularization (L)	Not performed
15	Spike–wave (L, central and temporal)	White matter damage	Not performed
16	Interhemispheric asymmetry	Right parietal and temporal leptomeningeal capillary malformation	Large cerebrospinal fluid spaces (R frontal)
17	No available EEG	Left volume loss, asymmetric myelination, parenchymal dystrophic calcifications, pial angioma	Not performed
18	Slow background activity (L, occipital)	Leptomeningeal capillary malformation (R, occipital and parietal)	Occipital calcifications (R)
19	Generalized spike–wave (R, occipital and parietal)	Normal	Not performed
20	Focal spikes (R, occipital)	Cortical–subcortical signal alteration (L)	Not performed
21	Focal spikes (R, occipital)	Normal	Not performed
22	Spikes	Multicystic encephalomalacia (L, temporal and frontal); cross atrophy; left Sylvian artery thrombosis	Significant findings
23	Generalized spike–wave (L–R, frontal and temporal)	Diffuse white matter hyperintensities; small cerebrospinal fluid cyst (L); corpus callosum hypoplasia	Not performed
24	Hypsarrhythmia and multifocal paroxysmal anomalies	Normal	Intraventricular hemorrhage (Grade II); periventricular hyperechogenicity
25	Normal	Cerebellar atrophy and hypoplasia	Not performed
26	Spikes	Normal	Not performed
27	Generalized spike–wave (L, temporal)	Normal	Not performed
28	Generalized spike–wave (L, temporal)	Normal	Not performed
29	Generalized spike–wave (L, occipital)	Normal	Not performed
30	Focal spikes (central and temporal)	Normal	Not performed
31	Multifocal paroxysmal anomalies	Dysmorphic ventricles; small corpus callosum; Galen vein malformation	Not performed
32	Focal spikes (L, central and temporal)	Normal	Not performed
33	Focal spikes (R, central and temporal)	Asymmetric lateral ventricles and temporal volume (L)	Not performed
34	Generalized spike–wave (L–R, central and temporal)	Cerebellar atrophy	Not performed
35	Generalized spike–wave (L, temporal)	Reduced lateral ventricle size	Increased ventricular spaces; left lateral ventricle mass
36	Generalized spike–wave (occipital)	Normal	Not performed
37	Generalized spike–wave (L–R, occipital)	Normal	Not performed
38	Normal	Normal	Not performed
39	Generalized spike–wave in all leads	Normal	Not performed

Legend: L: left; R: right;

### 3.4. Neuroimaging

**Figure 8:**
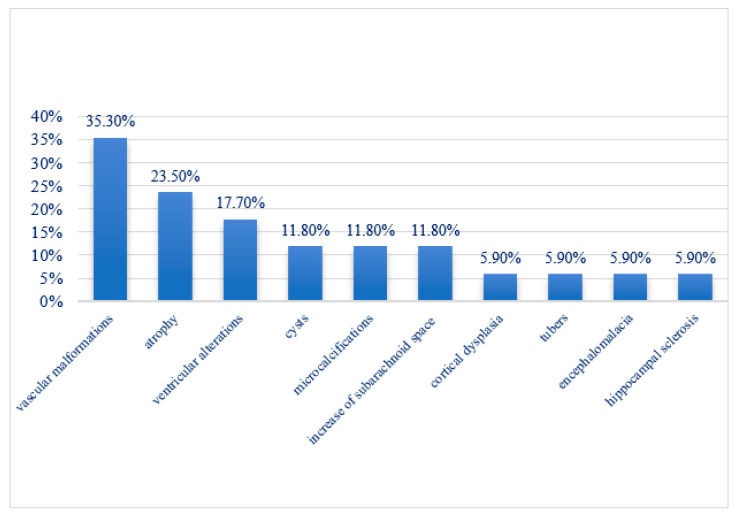
MRI findings of the patients.

Magnetic resonance imaging (MRI) identified focal structural abnormalities in 43.6% of patients (17/39) (Table 2). The most frequently detected pathologies included vascular malformations (35.3%), such as venous thrombosis, venous engorgement, and capillary malformations. Cerebellar, cortical, or corpus callosum atrophy was present in 23.5% of cases, while ventricular abnormalities were detected in 17.7%. Additional findings included microcalcifications, cystic formations, white matter changes, and increased subarachnoid space in 11.8% of cases. Less common but notable findings included cortical dysplasia, tubers, encephalomalacia, and hippocampal sclerosis, each present in 5.9% of cases (Figure 8).

In cases where MRI findings were unremarkable (22/39), EEG abnormalities most frequently localized to the central–temporal regions (40.9%), followed by the temporal (33.3%) and occipital (22.7%) regions. In contrast, both patients with frontal epilepsy (2/39) exhibited structural abnormalities on MRI. Computed tomography (CT) was performed in 15.4% (6/39) of cases, with 66.7% showing no abnormalities. When present, significant findings included intracranial calcifications and enlarged cerebrospinal fluid spaces. Brain ultrasound was utilized in 45.5% of infants under one year of age (5/11), with abnormal findings detected in 60% (3/5), including ventricular dilation and periventricular hyperechogenicity.

### 3.5. Treatment

**Figure 9:**
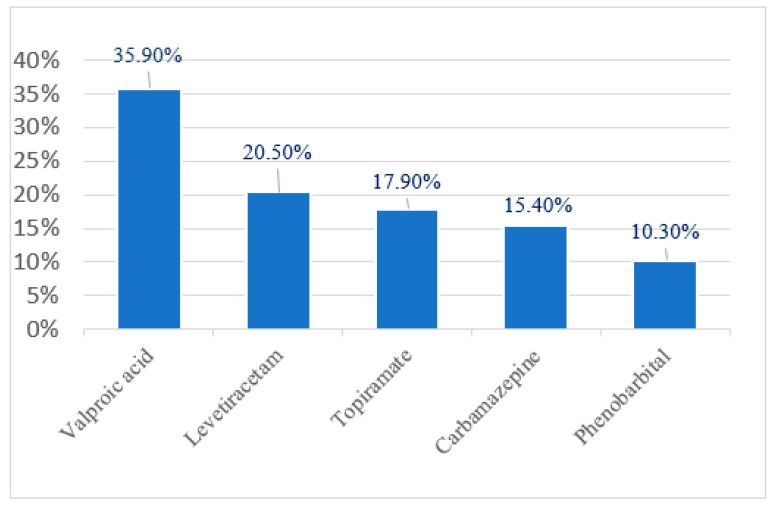
Medications used.

**Figure 10:**
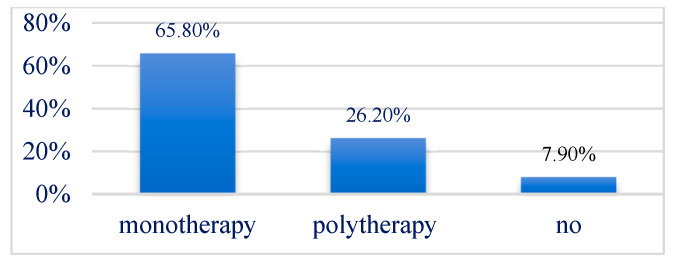
Treatment regimen employed in the patients.

An in-depth analysis of pharmacological treatment revealed that valproic acid was the most commonly used antiepileptic drug, prescribed in 35.9% of cases, with 57% of these patients receiving it in combination with other antiepileptic medications. Levetiracetam was administered in 20.5% of cases, with 75% of these patients receiving it as part of a polytherapy regimen. Topiramate was used in 17.9% of children, with 62.5% receiving it as monotherapy. Carbamazepine was prescribed in 15.4% of cases, with monotherapy accounting for 66.7% of these cases. Phenobarbital was administered in 10.3% of children, with three-quarters of these patients receiving it as monotherapy (Figure 9 and Figure 10).

Among the 39 patients, three (7.9%) had not yet initiated treatment. Monotherapy was administered in 25 patients (65.8%), whereas polytherapy was required in 10 patients (26.3%). Treatment data were unavailable for one patient (Table 2).

Only one patient (with mesial temporal sclerosis) was identified as a potential surgical candidate; however, surgery was not performed due to the parents’ refusal.

## 4. Discussion

This study is among the first to comprehensively integrate detailed genetic analysis, extensive neurophysiological assessments, and neuroimaging findings to provide novel insights into pediatric focal epilepsy. While previous research has focused on specific aspects of focal epilepsy, the multidisciplinary approach adopted in our study allows for a broader perspective on its clinical characteristics, diagnostic challenges, and treatment strategies.

The study cohort included 20 males (51.3%) and 19 females (48.7%), with a *p*-value of 0.82, confirming that gender does not significantly influence the onset of focal epilepsy. This finding aligns with previous literature, which has consistently demonstrated no gender predilection in focal epilepsy [4,5]. The absence of a gender-related difference in our study supports the hypothesis that genetic and environmental factors play a more critical role in epilepsy development than sex-specific influences. Seizure onset occurred after the first year of life in 71.8% of patients, with a highly significant *p*-value of <0.0001, reinforcing the well-established evidence that focal epilepsy primarily emerges in children older than one year [6]. This finding is critical in distinguishing focal epilepsy from generalized epilepsy syndromes, many of which manifest during infancy or early childhood. The later onset of focal epilepsy could be related to the progressive maturation of the cerebral cortex and neuronal networks, as well as environmental triggers, acquired brain insults, or genetic predisposition. Developmental disabilities were identified in 41% of patients, though the association was not statistically significant (*p* = 0.11). This result is in agreement with prior studies indicating that epilepsy itself does not necessarily correlate with motor dysfunction but is often associated with slowing of psychomotor processing [7]. The presence of cognitive impairment in some epilepsy patients is believed to result from abnormal cortical excitability, seizure burden, and underlying structural abnormalities rather than epilepsy per se [8,9]. A significant finding in our study was that the majority of patients had no family history of epilepsy or febrile seizures, a highly significant result (*p* = 0.0001). This suggests that most cases of pediatric focal epilepsy in our cohort were non-familial, possibly due to sporadic or mosaic mutations, prenatal risk factors, or acquired brain insults. However, the literature confirms that a positive family history is an important risk factor for epilepsy, particularly among siblings and offspring, although parental epilepsy is often underreported [10]. Regarding birth and delivery factors, our analysis found no significant difference between eutocic and dystocic deliveries (*p* = 0.182), in contrast with previous research that suggested a higher risk of epilepsy following perinatal complications [11,12,13]. However, prenatal risk factors were significantly associated with epilepsy onset compared to perinatal ones (*p* < 0.01), and postnatal risk factors were more significant than perinatal ones (*p* = 0.02). Among the most frequent risk factors identified were genetic mutations (20.5%), risk of miscarriage (17.8%), febrile seizures (15.4%), and asphyxia (12.8%), suggesting their potential contribution to epilepsy development. These findings underscore the importance of prenatal screening, genetic counseling, and early postnatal monitoring in at-risk children. A particularly notable finding was the high prevalence of cutaneous anomalies, observed in 80% of patients. Among these, 91.7% exhibited café-au-lait spots, and 25% had hypopigmented lesions. However, only 10.3% of patients were diagnosed with a neurocutaneous disorder, and none had neurofibromatosis type 1 (NF1) or neurofibromatosis type 2 (NF2). The presence of skin discolorations was highly significant (*p* < 0.0001), aligning with previous studies [14,15]. The association between cutaneous anomalies and epilepsy remains an area of interest, particularly in the context of undetected genetic syndromes that may present with both neurological and dermatological manifestations. Our study confirmed that temporal seizures were the most frequently observed type, consistent with prior studies [16]. However, the distribution of epileptic foci varied compared to other research [17]. Temporal seizures were frequently associated with a spike-and-wave (S&W) pattern, which was significantly more common (*p* < 0.005) than isolated spike discharges. Temporal seizures were also the most frequently associated with negative MRI findings (40.9%), showing significant differences when compared to multifocal or hypsarrhythmic patterns (*p* = 0.004 in both cases), though not significantly different from occipital seizures (*p* = 0.2). These findings emphasize that negative MRI findings should not exclude the diagnosis of epilepsy, particularly in cases where EEG confirms epileptiform discharges. The EEG remains the most sensitive diagnostic tool for evaluating epileptic activity, even in patients with normal structural neuroimaging. MRI was positive in 43.6% of cases, a proportion that varies across prior studies. J. et al. [18] reported a 40% prevalence of MRI abnormalities, but their study focused on children under two years of age, whereas estimates for older children range from 16% to 26% [19]. Eltze et al. [20] found positive MRI findings in 51% of cases, likely due to a younger patient cohort. In our study, vascular malformations were the most frequently detected cortical abnormality (35.8%), a finding that differs from studies where other structural lesions were more prevalent [21,22,23]. A significant difference was observed between vascular malformations and mesial sclerosis (*p* < 0.03), the latter being a more common lesion in adult epilepsy but relatively rare in pediatric cases [21]. A significant difference was observed in the diagnostic sensitivity of electroencephalography (EEG) compared to magnetic resonance imaging (MRI). The proportion of cases in which EEG yielded significant findings (89.7%) was substantially higher than that of MRI (43.6%), with a highly significant statistical difference (*p* < 0.001). This discrepancy became even more pronounced when evaluating cases in which both methods failed to provide significant findings (13.2% for EEG vs. 56.4% for MRI; *p* < 0.0001).

**Figure 11:**
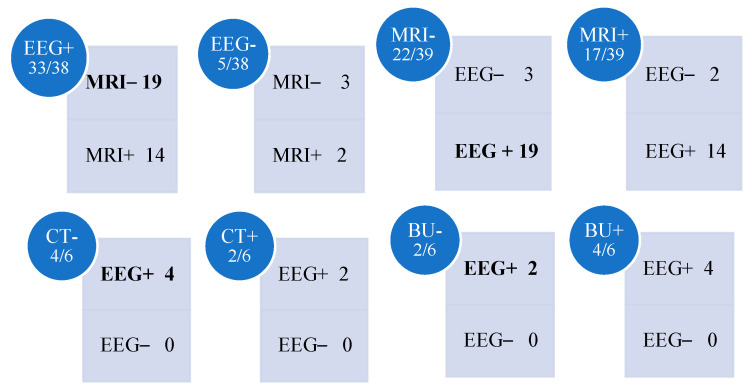
Matrices showing the relationships between the different diagnostic methods. Legend: BU: brain ultrasound. CT: brain computed tomography; EEG: electroencephalography MRI: brain magnetic resonance imaging.

Analysis of the data (Figure 11) consistently revealed high levels of significance regarding the diagnostic utility of EEG in comparison to MRI. Specifically, considering MRI-negative cases (22/39), the proportion of patients within this group who also had non-significant EEG findings was notably low (3/22), yielding a *p*-value < 0.001. This indicates that, due to its superior sensitivity, EEG enabled the diagnosis of focal seizures in 19 out of 22 children. Further comparison between EEG-positive cases (33/38) and MRI-positive cases within the same cohort (14/33) also resulted in a highly significant *p*-value (*p* < 0.0001). 

Although there were instances where EEG findings were non-significant, they remained statistically less frequent than MRI-negative cases: EEG-negative (5/38) versus MRI-negative (3/5), with a *p*-value < 0.01. Despite the superior sensitivity of EEG over MRI in diagnosing epilepsy, a notable subgroup of patients (12.8%) presented EEG-negative results, potentially due to limitations in EEG interpretation. Among these, three cases were also MRI-negative. Consequently, 7.7% of the patients (3/39) lacked diagnostic confirmation through either modality, relying solely on clinical manifestations. While clinical presentation remains pivotal in epilepsy diagnosis, the absence of an identifiable epileptogenic focus or structural abnormalities complicates understanding the pathology, therapeutic decision-making, and prognostic evaluation. Furthermore, the possibility of diagnostic errors, given the operator-dependent nature of both EEG and MRI, should be considered.

These findings reaffirm EEG as the most specific and sensitive diagnostic tool for epilepsy, in alignment with existing literature, despite the absence of direct comparative studies between EEG and MRI [16].

In an attempt to establish a statistical correlation between different EEG patterns and MRI findings, no statistically significant associations emerged (*p* = 0.76; *p* = 0.97). Specifically, seven out of seventeen (41.2%) MRI-positive cases were associated with a spike-and-wave (S&W) pattern, while three (17.7%) exhibited isolated spike discharges. Similarly, eight out of twenty-two (36.4%) MRI-negative cases displayed S&W activity, and four (18.2%) showed spike discharges, indicating that these EEG patterns were the most frequently observed regardless of MRI significance.

Analysis of epileptic foci in relation to MRI findings (Table 2) revealed that centrotemporal seizures, the most common seizure type in this cohort (41%), exhibited MRI-negative results in 56.3% of cases; however, this association was not statistically significant (*p* = 0.48), indicating that MRI positivity in these seizures remains unpredictable. Similarly, occipital seizures (20.5%) demonstrated a high rate of MRI-negative findings (62.5%), though without statistical significance (*p* = 0.33). Additionally, a direct comparison between temporal and occipital seizures yielded a *p*-value of 0.77, suggesting that MRI negativity is equally probable in these two regions. Interestingly, this contrasts with previous studies, which suggest that MRI-negative findings are more commonly observed in temporal rather than occipital seizures [24]. Conversely, MRI abnormalities were detected in all cases of frontal seizures (2/2), in agreement with the findings of Vecchi et al. [17].

When comparing EEG and computed tomography (CT), despite the lower number of required examinations, EEG remains the most reliable diagnostic tool. In cases where CT was negative (4/6), EEG consistently yielded significant findings (100%, *p* < 0.04). Similarly, a comparison between CT and MRI underscores the superior sensitivity of MRI, as corroborated by existing literature [24,25,26,27]. Notably, in cases where CT was non-significant, MRI was frequently positive, demonstrating its greater diagnostic yield.

Moreover, in cases where brain ultrasonography (BU) identified structural alterations (4/6), EEG findings were consistently significant. Even in BU-negative cases (2/6), EEG continued to demonstrate superior diagnostic accuracy.

In the literature, we find an association between MRI-negative findings and specific seizure types, particularly those of temporal origin [28,29]. One of the most common causes of temporal seizures is focal cortical dysplasia (FCD), which is often subtle and can be undetectable on conventional neuroimaging. It is likely that MRI-negative findings in our study are attributable to underlying cortical dysplasia that remains occult due to imaging limitations. Among patients with MRI-negative epilepsy, FCD is the most frequently identified pathology [30,31,32,33]. A prospective study [34] revealed that 65% of previously MRI-negative patients exhibited new lesions when re-examined, with FCD being present in nearly two-thirds of those initially classified as MRI-negative using 1.5T but later confirmed as positive with 3T imaging. This led to a change in clinical management in 38% of cases. Thus, MRI-negative epilepsy should not automatically be considered non-lesional. Multiple studies indicate that an average of 46% of cases initially classified as MRI-negative eventually reveal epileptogenic lesions [35,36,37]. A study by Bien et al. [38] analyzing 1192 patients reported that 16% (190 cases) were MRI-negative, with 29 undergoing surgical intervention; histopathological analysis revealed positive findings, predominantly FCD, in nine of these cases. Widjaja and Raybaud [39] argue that in some patients with intractable focal epilepsy, structural MRI may fail to demonstrate a lesion. However, when these patients undergo surgical resection, their outcomes tend to be poorer than those with identified lesions, and histological examination frequently uncovers subtle malformations of cortical development, such as microdysgenesis or gliosis. MRI may yield false-negative results due to the complexity of the brain’s convolutional pattern, making subtle lesions difficult to detect through conventional qualitative assessment. Additionally, studies in pediatric patients with refractory epilepsy suggest a high likelihood of FCD being the underlying pathology. Jay et al. [40] reported an FCD prevalence of 30%, while Farrell et al. [41] documented a 39% frequency in surgically resected cases. Recently, ultra-high-field MRI (7T and above) has garnered increasing interest for clinical applications. Several studies highlight the advantages of this technology, not only in research but also in improving diagnostics and patient management. A substantial proportion (approximately 25%) of surgical candidates show no detectable structural abnormalities on standard MRI scans. The implementation of higher-resolution imaging techniques is critical for improving surgical outcomes and optimizing presurgical planning, reducing reliance on invasive procedures such as intracerebral electrode implantation [42,43].

Intriguing aspects have been noted from functional MRI. In a recent analysis, the complexity of functional connectivity alterations in pediatric focal epilepsy has been highlighted, emphasizing both local and global network disruptions. The inconsistent patterns of increased and decreased function connectivity suggest dynamic compensatory mechanisms or variable disease progression. The involvement of interhemispheric connections and the default mode network aligns with our observations, reinforcing the role of large-scale network dysfunction in epilepsy. Understanding these connectivity changes is crucial for refining neuroimaging biomarkers and tailoring individualized therapeutic strategies in pediatric focal epilepsy [44].

A notable finding in our study pertains to the predominant use of valproate (VA), despite guideline recommendations [44,45] favoring its application primarily in generalized epileptic seizures. For focal seizures, first-line treatments should include carbamazepine (CBZ) or lamotrigine (LTG), with levetiracetam (LEV) or oxcarbazepine (OXC) as secondary options. VA should only be prescribed for newly diagnosed focal seizures when CBZ and LTG are ineffective or poorly tolerated, or as an adjunct therapy alongside other antiepileptic drugs (AEDs) such as CBZ, clobazam (CLB), gabapentin (GPT), LEV, LTG, OXC, topiramate (TPM), and zonisamide (ZNS).

Several studies analyzing treatment outcomes reinforce these therapeutic preferences. A 2018 Italian study assessed the comparative efficacy of AEDs in pediatric populations (0–18 years) by analyzing 46 randomized clinical trials encompassing 5652 individuals [46]. This meta-analysis determined that CBZ and LTG were the most effective treatments for newly diagnosed focal epilepsy. For refractory focal epilepsy, LEV and perampanel demonstrated greater efficacy compared to a placebo, while adrenocorticotropic hormone was the most effective treatment for infantile spasms. In contrast, ethosuximide (ETS) and VA were found to be superior to LTG for absence seizures, though LTG and LEV may still be viable alternatives, particularly in women of childbearing age [46]. Notably, VA is contraindicated in females of reproductive age unless alternative treatments are ineffective or intolerable due to its teratogenicity. Regulatory bodies such as the MHRA (March 2018) and AIFA (August 2018) issued guidelines restricting VA use in pregnancy, further reinforced in April 2020 [47,48]. Despite these recommendations, our study revealed that some female patients remain under VA treatment, comprising 23.5% (4/17) of the cohort (excluding two 19-year-old individuals not undergoing treatment or unaware of their regimen). A concerning finding is that VA was prescribed as the first-line treatment for focal seizures in 34.3% of cases, whereas LEV was first-line in 31.4%, and TPM in 8.6%.

These prescribing patterns deviate from established guidelines, potentially due to VA’s broad-spectrum efficacy, which may make it a preferred option in cases where the clinical or electrophysiological diagnosis remains unclear. Similarly, LEV, another broad-spectrum AED, may be favored as it can be used for both focal and generalized seizures.

Lastly, patients with focal epilepsy who become drug-resistant should promptly undergo assessment in a center for surgical treatment of epilepsy. After excluding pseudo-resistance (i.e., inadequate drug dosage or wrong therapeutic choices), these patients should be considered for presurgical evaluation as a means to identify the location and extent of the epileptogenic zone and assess their candidacy for a surgical procedure [48]. A surgical perspective was considered, but no patients in this cohort underwent surgical treatment. Only one patient (with mesial temporal sclerosis) was identified as a potential surgical candidate; however, surgery was not performed due to the parents’ refusal.

## 5. Study Limitations

The interpretation of EEG and imaging results is highly operator-dependent. Consequently, there is a possibility that we may have missed, underestimated, or overestimated certain clinical findings in our patients. Additionally, the lack of more advanced diagnostic tools, including high-resolution MRI (3–7 Tesla), may have limited the accuracy and depth of our assessments. Furthermore, the manual nature of data collection and analysis introduces an inherent risk of human error, despite our best efforts to maintain accuracy and consistency.

## 6. Conclusions

This study provides a comprehensive analysis of the clinical, diagnostic, and therapeutic aspects of focal epileptic seizures in pediatric patients. Among 39 recruited patients (51.3% males, 48.7% females), the mean age at seizure onset was 4.8 years, predominantly occurring after the first year of life (*p* < 0.0001). Family history did not appear to be a strong determinant, with only 23.3% of cases reporting a familial history of epilepsy. Prenatal (46.2%) and postnatal (43.6%) risk factors were significant contributors to epilepsy onset, with genetic mutations (20.5%), risk of miscarriage (17.8%), febrile seizures (15.4%), and asphyxia (12.8%) being the most prevalent (*p* < 0.01 and *p* = 0.02, respectively). Cutaneous anomalies, particularly cafè-au-lait spots (91.7%), were significantly associated with epilepsy (*p* < 0.0001). Developmental disabilities were observed in 41% of cases, correlating with MRI abnormalities (*p* = 0.0009). Temporal lobe involvement was most common (42.1%), predominantly presenting with a spike–wave EEG pattern (*p* < 0.005). MRI abnormalities were identified in 43.6% of cases, with vascular alterations (35.8%) being most frequent, while hippocampal sclerosis remained rare in pediatric cases. EEG demonstrated superior diagnostic sensitivity (89.7%) over MRI (*p* < 0.001), and in MRI-negative cases, EEG identified the epileptogenic focus in 86.4% of children.

This study highlights the need for advanced imaging techniques, as MRI-negative cases may still harbor lesions such as focal cortical dysplasia. Higher-resolution MRI (3T or 7T) could significantly improve epilepsy diagnostics and surgical planning. However, such techniques are not currently available in many parts of the world, given their high costs. Regarding treatment, valproate (VA) was the most used drug (36.8%), despite guidelines recommending it primarily for generalized seizures. CBZ, the preferred first-line drug for focal epilepsy, was underutilized (15.8%). VA was also prescribed to 23.5% of female patients despite known teratogenic risks. These findings underscore the need for improved adherence to treatment guidelines to enhance patient outcomes.

## Figures and Tables

**Figure 7:**
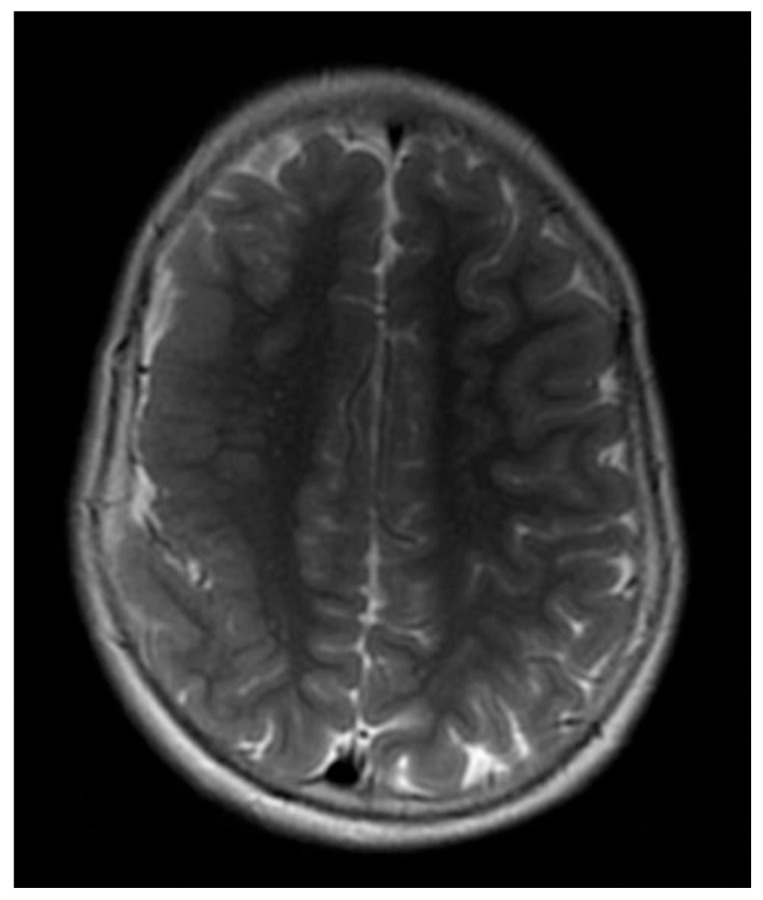
A 1.5 Tesla, T2-weighted axial scan of diffuse polymicrogyria which involves the parasagittal regions of both hemispheres and the right fronto-parietal cortex, which also appear thickened and dysplastic (Patient 13).

**Table 1:** Clinical aspects of the patient with seizures.

Patient	Age at Onset	Physical Features	Seizure Semiology	Psychomotor Development
1	4 years	None	Focal impaired awareness seizures with LoC, Si, VF	Normal
2	4 months	Single café-au-lait macule (lumbar region)	Focal seizures with head deviation and bilateral eyelid ptosis	Normal
3	3 years	Hypopigmented macules (back and limbs)	Focal seizures with eyelid and perioral myoclonus, gelastic seizures, leading to LoC	Delayed, ADHD
4	5 months	Single café-au-lait macule, hypotonia of lower limbs, unilateral hypertonia (R)	Clonic seizures affecting the upper limbs	Normal
5	3 years	None	Generalized seizures with visual fixation, atonic features, and clonic jaw movements (~30 min)	Normal
6	1 year	None	Focal seizures with VF, recurrent sialorrhea, dysarthria, hypoesthesia of limbs, urinary incontinence (~1 min)	Delayed
7	3 years	None	Autonomic seizures with pallor, diaphoresis, hypotonia (~1 min), swallowing difficulties, and impaired awareness	Normal
8	5 years	Hyperreflexia	Focal seizures with impaired awareness, uncertain gait, VF, focal tonic seizures in the right upper limb, progressing to GTCS	Normal
9	9 years	Single café-au-lait macule (left hemithorax)	Focal clonic seizures of the lower limb (R), evolving into GTCS, ocular version, and LoC	Normal
10	10 years	None	Focal seizures with impaired awareness, buccal paresthesia, unilateral clonus, ocular version, recurrent vomiting, and postictal headache	Normal
11	12 years	Three café-au-lait macules	Generalized seizures with left deviation, sialorrhea, and clonic activity of all four limbs, impaired awareness	Delayed
12	9 years	None	Generalized tonic–clonic seizures (GTCS), hypertonia, postictal headache, and vomiting	Normal
13	3 months	Mild hypotonia	Head and eye deviation (R), buccal automatisms, and tonic movements of the right upper limb	Delayed
14	11 years	None	Focal clonic seizures affecting the upper limb, VF	Normal
15	12 years	Two café-au-lait macules	Gelastic seizures, LoC, head deviation, sialorrhea, clonic movements in four limbs, impaired awareness	Normal
16	5 months	Angioma in trigeminal branches and right trunk	Head and eye deviation (L), transient ischemic attack (TIA) with hemiparesis, prolonged tonic–clonic seizures	Delayed
17	2 years	Eyelid and left-sided facial angioma, iris angioma	No seizures reported	Normal
18	3 years	Facial angioma, nasal root (R)	TIA with lameness in lower limb (R), headache in orbital and temporal region (R), vomiting	Delayed
19	6 years	None	Déjà-vu aura tachycardia, headache, gastralgia, confusion	Normal
20	4 years	Single café-au-lait macule, reduced reflexes bilaterally, dysarthria, lunar facies	Paroxysmal chewing movements, sialorrhea, buccal deviation (L), generalized hypertonia (R), LoC, VF	Delayed
21	3 years	Subareolar café-au-lait macule (R)	Hypertonia, sialorrhea, LoC, clonus	Delayed
22	4 years	Hemiplegia (R)	Unknown	Delayed
23	1 year	Hypotonic muscle mass	Tremors, buccal deviation, LoC, clonic jaw movements, sialorrhea	Delayed
24	9 months	Microcephaly, axial hypotonia, arthrogryposis, single palmar sulcus, hyperreflexia	Perioral cyanosis, ocular version (~30 sec)	Delayed
25	10 months	Hemimegalencephaly (R), reduced reflexes	Hypotonia, VF	Delayed
26	11 months	Two café-au-lait macules, bilateral cavus foot	Unknown	Delayed
27	2 years	Hypochromic spots, three café-au-lait macules (R)	Unknown	Delayed
28	5 years	None	No seizures reported	Normal
29	9 years	None	Headache, autonomic seizures with sudden pallor, dizziness, hypotonia (~10 min), weakness	Normal
30	4 months	Microcephaly	Generalized hypertonia, upwards gaze deviation	Normal
31	7 months	Microcephaly, bilateral hyperreflexia, hypertonia (four limbs)	Multifocal seizures with impaired awareness	Delayed
32	3 years	Hyposthenic right upper limb	Head and eye deviation (R), hypertonicity (R upper limb), LoC, eyelid myoclonus	Normal
33	9 years	None	Lip deviation (L), drooling, VF, eyelid myoclonus, LoC, postictal weakness and paraesthesia (R)	Normal
34	2 years	Multiple café-au-lait macules	VF, sialorrhea, hypertonia, subsequent tonic–clonic contractions, opisthotonus, ocular version, bruxism	Delayed
35	8 years	None	Sialorrhea, chewing movements, generalized tremors, buccal rim and gaze deviation (R), eyelid blinking, impaired awareness	Normal
36	4 years	Single café-au-lait macule, hyperexcitable patellar reflex	Cyanosis, loss of balance, GTCS, sialorrhea, LoC, profound drowsiness, fear-induced seizures, mastication, bruxism	Normal
37	12 years	None	VF, sialorrhea, hypertonia, subsequent tonic–clonic contractions, opisthotonus, ocular version, bruxism	Normal
38	13 years	None	VF	Normal
39	11 years	Hypochromic macule	Right focal seizures with impaired awareness	Normal

Legend: ADHD: Attention Deficit Hyperactivity Disorder; GTCS = Generalized Tonic-Clonic Seizures; L = left; LoC = loss of consciousness; R = right; S = seizure; Si = sialorrhea; VF = visual fixation; TIA: transitory ischemic attack.

## Data Availability

Data supporting reported results can be found in the archives of the University of Catania (www.unict.it), accessed on 12 February 2025 or may be requested from the corresponding authors by email (andrea.pratico@unikore.it).

## Supplementary Materials

The following supporting information can be downloaded at: https://www.mdpi.com/article/10.3390/jcm14072234/s1, Table S1: STROBE Statement—Checklist of items that should be included in reports of cohort studies.

## References

[1] Fisher R.S., Acevedo C., Arzimanoglou A., Bogacz A., Cross J.H., Elger C.E., Engel J., Forsgren L., French J.A., Glynn M. (2014). ILAE Official Report: A practical clinical definition of epilepsy. Epilepsia.

[2] Forsgren L., Beghi E., Õun A., Sillanpää M. (2005). The epidemiology of epilepsy in Europe—A systematic review. Eur. J. Neurol..

[3] Laoprasert P. (2011). Atlas of Pediatric EEG.

[4] Reddy D.S. (2017). The neuroendocrine basis of sex differences in epilepsy. Pharmacol. Biochem. Behav..

[5] Carlson C., Dugan P., Kirsch H.E., Friedman D., EPGP Investigators (2014). Sex differences in seizure types and symptoms. Epilepsy Behav..

[6] Ochoa-Gómez L., López-Laso E., Luna-Muñoz E., Rodrigo C.F., Martínez R.F., Samper-Villagrasa P., Monge-Galindo L., Peña-Segura J.L., García-Jiménez M.C. (2017). A study of epilepsy according to the age at onset and monitored for 3 years in a regional reference paediatric neurology unit. An. Pediatr..

[7] Boelen S., Lodder S.S., Berends A., Veldwijk H., van de Ven-Verest M., Tan I.Y., Aldenkamp A.P. (2005). Effect of epilepsy on psychomotor function in children with uncomplicated epilepsy. Dev. Med. Child Neurol..

[8] Vlooswijk M.C.G., Jansen J.F.A., Majoie H.J.M., de Krom M.C., Majoie H.J., Hofman P.A., Aldenkamp A.P., Backes W.H. (2011). Loss of network efficiency associated with cognitive decline in chronic epilepsy. Neurology.

[9] Helmstaedter C., Witt J.A. (2017). Epilepsy and cognition—A bidirectional relationship?. Seizure.

[10] Ottman R., Hirose S., Jain S., Vasoli V.M., Hauser W.A., Buchhalter J.R. (2011). Accuracy of family history information on epilepsy and other seizure disorders. Neurology.

[11] Demissie K., Breckenridge M.B., Rhoads G.G., Balasubramanian B.A., Gandhi K., Joseph K.S., Kramer M. (2004). Operative vaginal delivery and neonatal and infant adverse outcomes: Population-based retrospective analysis. Br. Med. J..

[12] Shakeshaft A., Panjwani N., McDowall R., Crudgington H., Peña Ceballos J., Andrade D.M., Beier C.P., Fong C.Y., Gesche J., Greenberg D.A. (2021). Trait impulsivity in Juvenile Myoclonic Epilepsy. Ann. Clin. Transl. Neurol..

[13] Laughon S.K., Berghella V., Reddy U.M., Sundaram R., Lu Z., Hoffman M.K. (2014). Neonatal and maternal outcomes with prolonged second stage of labor. Obstet. Gynecol..

[14] Ruggieri M., Polizzi A., Catanzaro S., Bianco M.L., Praticò A.D., Di Rocco C. (2020). Neurocutaneous melanocytosis (melanosis). Childs Nerv Syst..

[15] Kakkar S., Mendiratta V., Sharma N., Aneja S., Harjai B. (2007). Cutaneous manifestations of seizure disorder in children—A study of 100 seizure patients. Pediatr. Dermatol..

[16] Noachtar S., Rémi J. (2009). The role of EEG in epilepsy: A critical review. Epilepsy Behav..

[17] Vecchi M., Baroni G., Brinciotti M., Stivala M., Guerrini R., Albamonte E., Ranalli D., Battaglia D., Lunardi G., Boniver C. (2016). Symptomatic and presumed symptomatic focal epilepsies in childhood: An observational, prospective multicentre study. Epilepsia.

[18] Coryell J., Gaillard W.D., Shellhaas R.A., Grinspan Z.M., Wirrell E.C., Knupp K.G., Wusthoff C.J., Keator C., Sullivan J.E., Loddenkemper T. (2018). Neuroimaging of early life epilepsy. Pediatrics.

[19] Berg A.T., Vickrey B.G., Langfitt J.T., Fulbright R.K., DiMario F., Testa F.M., Levy S.R. (2009). Frequency, prognosis, and surgical treatment of structural abnormalities seen with magnetic resonance imaging in childhood epilepsy. Brain.

[20] Eltze C.M., Chong W.K., Cox T., Whitney A., Cortina-Borja M., Chin R.F., Scott R.C., Cross J.H. (2013). A population-based study of newly diagnosed epilepsy in infants. Epilepsia.

[21] Wang P.J. (1997). Magnetic resonance imaging in symptomatic/cryptogenic partial epilepsies of infants and children. Acta Paediatr. Sin..

[22] Kim D.W., Lee S.Y., Chung S.E., Cheong H.K., Jung K.Y., Korean Epilepsy Society (2014). Clinical characteristics of patients with treated epilepsy in Korea: A nationwide epidemiologic study. Epilepsia.

[23] Kramer U. (2008). Atypical presentations of benign childhood epilepsy with centrotemporal spikes: A review. J. Child Neurol..

[24] Ben Ameur S., Bouguila H., Mhirsi A., Yaich S., Mnif Z., Kamoun T., Hachicha M. (2014). Cerebral imaging in epileptic children: Study of 140 cases. Tunis. Med..

[25] Bronen R.A., Fulbright R.K., Spencer D.D., Spencer S.S., Kim J.H., Lange R.C., Sutilla C. (1996). Refractory epilepsy: Comparison of MR imaging, CT, and histopathologic findings in 117 patients. Radiology.

[26] Bradley W.G., Shey R.B. (2000). MR imaging evaluation of seizures. Radiology.

[27] Ali A., Akram F., Khan G., Hussain S. (2017). Paediatrics brain imaging in epilepsy: Common presenting symptoms and spectrum of abnormalities detected on MRI. J. Ayub Med. Coll. Abbottabad.

[28] Muhlhofer W., Tan Y.L., Mueller S.G., Knowlton R. (2017). MRI-negative temporal lobe epilepsy—What do we know?. Epilepsia.

[29] Lee Y.J., Lee J.S. (2013). Temporal lobe epilepsy surgery in children versus adults: From etiologies to outcomes. Korean J. Pediatr..

[30] Wong-Kisiel L.C., Britton J.W., Patterson M.C., Witte R.J., Santana-Almansa A., Worrell G.A., Britton J., Brinkmann B.H. (2018). Morphometric analysis on T1-weighted MRI complements visual MRI review in focal cortical dysplasia. Neurology.

[31] Wang Z.I., Alexopoulos A.V., Jones S.E., Jaisani Z., Najm I.M., Prayson R.A. (2013). The pathology of magnetic-resonance-imaging-negative epilepsy. Mod. Pathol..

[32] Boutzoukas E.M., Stein M.A., Luberto C.M., Sepeta L.N., You X., Gaillard W.D., Wallace G.L., Berl M.M. (2020). Cortical thickness in childhood left focal epilepsy: Thinning beyond the seizure focus. Epilepsy Behav..

[33] Madan N., Grant P.E. (2009). New directions in clinical imaging of cortical dysplasias. Epilepsia.

[34] Bartolini L., Aronica E., Baayen J.C., Sepeta L.N., Oluigbo C.O., Havens K., Freilich E.R., Schreiber J.M., Gaillard W.D. (2017). Temporal lobe epilepsy and focal cortical dysplasia in children: A tip to find the abnormality. Epilepsia.

[35] Alarcón G., Binnie C.D., Elwes R.D., Selway R.P., Lacruz M.E., Elwes R.D., Jarosz J.M., Honavar M., Brunhuber F., Mullatti N. (2006). Is it worth pursuing surgery for epilepsy in patients with normal neuroimaging?. J. Neurol. Neurosurg. Psychiatry.

[36] Fong J.S., Englot D.J., Yang L., Prayson R.A., Busch R., Bingaman W. (2011). Seizure outcome and its predictors after temporal lobe epilepsy surgery in patients with normal MRI. Epilepsia.

[37] Cukiert A., Burattini J.A., Marquez R.M., Sousa A., Vieira J.O., Argentoni M., Forster C., Baldauf C. (2001). Results of surgery in patients with refractory extratemporal epilepsy with normal or nonlocalizing magnetic resonance findings investigated with subdural grids. Epilepsia.

[38] Bien C.G., Schulze-Bonhage A., Deckert J., Clusmann H., Becker A.J., Urbach H. (2009). Characteristics and surgical outcomes of patients with refractory magnetic resonance imaging-negative epilepsies. Arch. Neurol..

[39] Widjaja E., Raybaud C. (2008). Advances in neuroimaging in patients with epilepsy. Neurosurg. Focus.

[40] Jay V., Becker L.E., Blaser S., Hwang P.A., Hoffman H.J., Harwood-Nash D. (1993). Pathology of temporal lobectomy for refractory seizures in children. J. Neurosurg..

[41] Farrell M.A., DeRosa M.J., Curran J.G., Secor D.L., Cornford M.E., Comair Y.G., Peacock W.J., Shields W.D., Vinters H.V. (1992). Neuropathologic findings in cortical resections (including hemispherectomies) performed for the treatment of intractable childhood epilepsy. Acta Neuropathol..

[42] Barisano G., Fattori S., Castellaro M., Jann K., Cabeen R., Wang D.J., Toga A.W., Law M. (2006). Clinical 7T MRI: Are we there yet? A review about magnetic resonance imaging at ultra-high field. Magn. Reason. Imaging.

[43] Trattnig S., Bogner W., Chmelik J., Hangel G., Strasser B., Dymerska B., Cardoso P.L., Robinson S.D. (2018). Key clinical benefits of neuroimaging at 7 T. Neuroimage.

[44] Feng X., Piper R.J., Prentice F., Clayden J.D., Baldeweg T. (2024). Functional brain connectivity in children with focal epilepsy: A systematic review of functional MRI studies. Seizure.

[45] Nosadini M., Eyre M., Giacomini T., Valeriani M., Della Corte M., Praticò A.D., Annovazzi P., Cordani R., Cordelli D.M., Crichiutti G. (2022). Early Immunotherapy and Longer Corticosteroid Treatment Are Associated With Lower Risk of Relapsing Disease Course in Pediatric MOGAD. Neurol. Neuroimmunol. Neuroinflammation.

[46] Nevitt S.J., Sudell M., Cividini S., Marson A.G., Smith C.T. (2017). Antiepileptic drug monotherapy for epilepsy: A network meta-analysis of individual participant data. Cochrane Database Syst. Rev..

[47] Rosati A., Ilvento L., Lucenteforte E., Pugi A., Crescioli G., McGreevy K.S., Virgili G., Mugelli A., De Masi S., Guerrini R. (2018). Comparative efficacy of antiepileptic drugs in children and adolescents: A network meta-analysis. Epilepsia.

[48] Nascimento F.A., Friedman D., Peters J.M., Bensalem-Owen M.K., Cendes F., Rampp S., Wirrell E., Blümcke I., Tatum W., Beniczky S. (2023). Focal epilepsies: Update on diagnosis and classification. Epileptic Disord..

